# Relationships among Dioxin-like Mitochondria Inhibitor Substances (MIS)-Mediated Mitochondria Dysfunction, Obesity, and Lung Function in a Korean Cohort

**DOI:** 10.3390/toxics12100735

**Published:** 2024-10-11

**Authors:** Hoonsung Choi, Kyungho Ha, Jin Taek Kim, Min Kyong Moon, Hyojee Joung, Hong Kyu Lee, Youngmi Kim Pak

**Affiliations:** 1 Department of Internal Medicine, Chung-Ang University College of Medicine, Seoul 06974, Republic of Korea; hoonsung@cauhs.or.kr; 2 Department of Food Science and Nutrition, Jeju National University, Jeju 63243, Republic of Korea; kyungho.ha@jejunu.ac.kr; 3 Department of Internal Medicine, Division of Endocrinology and Metabolism, Nowon Eulji University Hospital, Eulji University School of Medicine, Seoul 01830, Republic of Korea; jtkimmd@eulji.ac.kr; 4 Department of Internal Medicine, Seoul Metropolitan Government Seoul National University Boramae Medical Center, Seoul 07061, Republic of Korea; mkmoon@snu.ac.kr; 5 Department of Public Health, Graduate School of Public Health, Seoul National University, Seoul 08826, Republic of Korea; hjjoung@snu.ac.kr; 6 Department of Internal Medicine, Seoul National University College of Medicine, Seoul 03087, Republic of Korea; hongkyu414@gmail.com; 7 Biomedical Science Institute, Department of Physiology, College of Medicine, Kyung Hee University, Seoul 02447, Republic of Korea

**Keywords:** obesity, lung function, mitochondrial dysfunction, MIS, structural equation modeling, dioxin-like chemicals, sex differences

## Abstract

Mitochondrial dysfunction is closely linked to obesity and diabetes, with declining lung function in aging increasing diabetes risk, potentially due to elevated serum levels of dioxin-like mitochondria inhibitor substances (MIS) from prolonged exposure to environmental pollutants. However, the mechanisms connecting MIS, mitochondria, lung function, and metabolic disorder remain unclear. In this study, we analyzed data from 1371 adults aged 40–69 years in the 2008 Korean Genome Epidemiologic Study (KoGES) Ansung cohort. We indirectly estimated dioxin-like MIS levels by measuring intracellular ATP (MIS_ATP_) and reactive oxygen species (MIS_ROS_) in cultured cells treated with the serum of participants. Using correlation analysis and structural equation modeling (SEM), we explored the relationships among MIS, mitochondrial function, body mass index (BMI), and lung function (FEV1 and FVC). Our findings revealed that MIS_ATP_ was associated with BMI in females and with FVC in males, while MIS_ROS_ correlated with both BMI and FVC in males, not in females. Significant associations between BMI and FVC were found in the highest MIS subgroup in both sexes. SEM analyses demonstrated that MIS negatively influenced mitochondrial function, which in turn affected BMI and lung function. Age-related declines in lung function were also linked to mitochondrial dysfunction. This study underscores the potential of MIS assays as alternatives for assessing mitochondrial function and highlights the importance of mitochondrial health in metabolic disorders and lung function.

## 1. Introduction

Mitochondrial dysfunction is a critical factor in energy metabolism and closely related to insulin resistance, type 2 diabetes, and metabolic syndrome (MetS) [1,2,3]. A decline in mitochondrial function and exposure to environmental polluting chemicals (EPCs), including persistent organic pollutants (POPs) and endocrine-disrupting chemicals (EDCs), are increasingly recognized as strong risk factors or predictors for type 2 diabetes, obesity, and MetS [4,5,6]. Most toxic effects of EPCs are mediated through the aryl hydrocarbon receptor (AhR) nuclear receptor, which is activated by a diverse range of AhR ligands, namely dioxins and dioxin-like chemicals. Some EPCs, such as dioxins and polychlorinated biphenyls (PCBs), are also reported to inhibit mitochondrial function through AhR-dependent (dioxin-like) and/or AhR-independent (non-dioxin-like) pathways [7]. Elevated serum levels of AhR ligands and mitochondria inhibitor substances (MIS) were observed in individuals with diabetes and MetS in Korean [5], Swedish [7,8], and Brazilian populations [9].

Serum levels of AhR ligands and MIS were indirectly estimated by measuring the AhR-dependent luciferase activity (AhRL), intracellular ATP (MIS_ATP_), and reactive oxygen species (ROS) (MIS_ROS_) after treating cultured cells with the patient serum [4]. Elevated serum MIS and AhRL levels in individuals with diabetes and MetS reflect prolonged and cumulative exposure to EPCs, including dioxin-like and/or non-dioxin-like chemicals [5]. In fact, certain dioxins, dioxin-like chemicals, and non-dioxin-like chemicals were detected in the serum of elderly Swedish participants in the PIVUS cohort [7]. The total toxic equivalence (TEQ) of these chemicals was positively correlated with AhRL and MIS_ROS_ and negatively with MIS_ATP_. Thus, both AhRL and MIS levels represent the contamination by EPCs, while MIS also indirectly reflects mitochondrial function in patients. It should be noted, however, that AhRL is not identical to MIS_ROS_ or MIS_ATP_, as some EPCs can affect intracellular ATP and ROS levels regardless of their dioxin-like properties.

Lung function is essential for whole-body energy metabolism because its main function is to provide oxygen and remove carbon dioxide for energy production in mitochondria. Indeed, lung functional status correlates with factors such as age, sex, body size (particularly height), muscle mass, physical activity [10], and more importantly, mitochondrial function [11]. Given the decline in metabolic rate with aging, age-related declines in lung function might be associated with mitochondrial dysfunction [12,13]. Our previous study reported that decreased lung function was associated with a higher risk of diabetes, though the underlying mechanism was not elucidated [14]. Based on these findings, we hypothesized that MIS in serum could contribute to the association between lung function and obesity.

In this study, we analyzed the relationships among lung function, obesity, and mitochondrial function in multifactorial disease models, utilizing serum AhRL and MIS levels (MIS_ATP_ and MIS_ROS_) previously analyzed in the Ansung cohort of the Korean Genome Epidemiologic Study (KoGES), a well-established community-based cohort study [4,5,15]. We employed structural equation modeling (SEM) to examine complex associations involving multiple factors, offering the advantage of constructing disease models based on existing knowledge [16,17]. We developed a framework where mitochondrial function, that is, MIS, serves as the central component linking body mass index (BMI) and lung function, demonstrating statistical relevance.

## 2. Materials and Methods

### 2.1. Participants

**Figure 1:**
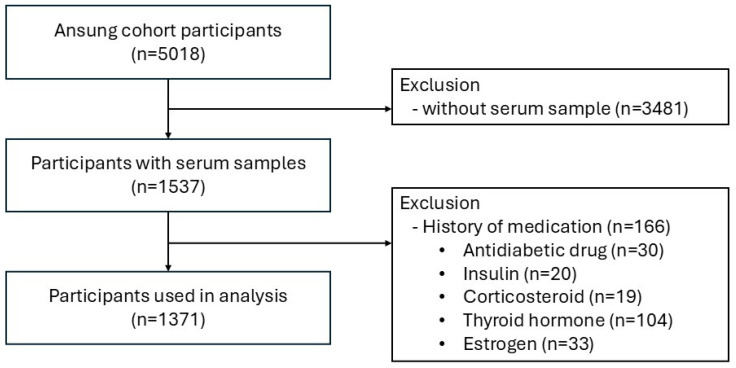
Flow chart for the selection of this study population.

This study analyzed clinical data and serum samples from the 2008 KoGES Ansung cohort, extending previously published research on this cohort [15]. The KoGES Ansung cohort was established to investigate the genetic and environmental etiology of common, complex diseases in Koreans [15,18]. Ansung, a representative rural farming community, had a population of 132,906 in 2000. Conducted between 2001 and 2003, the Ansung cohort included 5018 of 7192 eligible adults, primarily farmers aged 40–69 years (response rate = 69.6%), with biennial follow-up surveys until 2020. Eligibility criteria at baseline included an age of 40–69 years, residence within the survey area for at least 6 months before testing, and sufficient mental and physical ability to participate. We utilized data from 1537 serum samples collected in earlier research focused on diabetes development, with sample selection aimed at maximizing statistical power based on glucose tolerance status [5]. Lung function tests were conducted throughout this study period as previously reported [19]. Among the 1537 participants, 166 individuals who had taken antidiabetic drugs or insulin, or hormone replacement therapy with corticosteroids, thyroid hormone, or estrogen were excluded from our analysis. Figure 1 presents the flow chart for the selection of this study population (n = 1371). This study protocol was approved by the Institutional Review Board of the Eulji University Hospital (IRB approval number: EMCS 2019-05-008) and the Korean Centers for Disease Control (approval number: 2019-EPI-41), and was conducted in accordance with the principles of the Declaration of Helsinki.

### 2.2. Assessment of Clinical Characteristics and Biological Parameters

All methods and the relevant results of the KoGES have been previously documented [15]. Briefly, medical histories, medication use, and various health-related variables were collected. Blood samples were analyzed in central laboratories for standard biochemical studies. Fasting plasma glucose, total cholesterol (TC), triglycerides (TG), and high-density lipoprotein (HDL) cholesterol were measured using a Hitachi 747 chemistry analyzer (Hitachi Ltd., Tokyo, Japan). Low-density lipoprotein (LDL) cholesterol was calculated using the Friedewald equation (LDL = TC – HDL − TG/5, mg/dL). The glycosylated hemoglobin (HbA1c) was measured by high-performance liquid chromatography (Variant II; BioRad Laboratories, Hercules, CA, USA) and the plasma insulin concentration by radioimmunoassay (Linco kit, St. Charles, MO, USA). Homeostasis model assessment of insulin resistance index (HOMA-IR) was calculated as follows: HOMA-IR = (fasting glucose [mmol/L]) × (fasting insulin [μU/mL])/22.5 [20]. Participants were categorized by smoking status (never-smokers, ex-smokers, and current smokers) and alcohol intake (never-drinkers, ex-drinkers, and current drinkers). Exercise levels were classified as non-exercisers, those exercising 1–2 times per week, and those exercising more than 3 times per week.

### 2.3. Pulmonary Function Tests

Pulmonary function tests were performed by a trained technician using a Vmax-2130 spirometer (Sensor Medics, Yorba Linda, CA, USA) according to the American Thoracic Society standard protocol [21]. Calibration and quality control of the spirometry tests were conducted as per guidelines. All participants underwent pulmonary function testing in a fasting state after rest in the morning. In this study, unadjusted raw values of forced vital capacity (FVC) and forced expiratory volume in 1 s (FEV_1_) were analyzed to assess the effects of age and sex on pulmonary function as covariates.

### 2.4. Serum AhRL, MIS_ATP_, and MIS_ROS_ Assays

AhRL levels in the serum were determined using a cell-based AhR ligand activity (CALA) assay [8]. Briefly, the pGL4-DRE-luc/pRL-mTK double-positive Hepa1c1c7 stable cells were cultured for 24 h with 10% heat-inactivated serum samples or charcoal-stripped human serum (control, CSS). Luciferase activities were measured using a Dual-Glo Luciferase assay system (Promega, Madison, WI, USA) and a luminometer (Berthold, Bad Wildbad, Germany). All assays were conducted in duplicate. AhRL values were converted to 2,3,7,8-tetrachlorodibenzodioxin (TCDD) equivalents (TCDDeq, pM) using a standard curve (0–10 pM of TCDD), prepared using the cells exposed to serially diluted TCDD (0–50 pM) for 24 h in the presence of 10% CSS. The intra- and interassay coefficients of variation of these methods were less than 5.0%.

MIS levels in the serum were determined by measuring intracellular ATP content (MIS_ATP_) and ROS generation (MIS_ROS_) [4,5]. Briefly, Hepa1c1c7 cells transfected with pRL-mTK were cultured for 24 h with serum or control samples. ATP content was determined using the CellTiter-Glo luciferase kit (Promega, Madison, WI, USA), normalized to Renilla luciferase activity. ROS level was determined using 5-(and-6)-chloromethyl-2′,7′-dichlorodihydrofluorescein diacetate and acetyl ester (CM-H_2_DCFDA; Molecular Probes, Eugene, OR, USA). Both MIS_ATP_ and MIS_ROS_ were expressed as a percentage of CSS-treated control. Intra- and inter-assay variation for these methods was less than 6.0%.

### 2.5. Structural Equation Modeling

Structural equation modeling (SEM) was employed to elucidate the causal connections among the factors. SEM analysis involved constructing a hypothetical framework based on existing theoretical knowledge and assessing model fitness. SEM is a comprehensive statistical technique used to analyze the structural relationship between observed (measured) variables and unobserved (latent) variables that could be essential concepts in the association network to explain the underlying mechanism. Two variables, ‘mitochondrial inhibition’ and ‘mitochondrial function’ were included as latent variables. Model fit was assessed using RMSEA (the root mean square error of approximation) and CFI (comparative fit index), with RMSEA values below 0.06 and CFI values exceeding 0.95 considered reasonable [22]. RMSEA assesses how far a hypothesized model deviates from a perfect model, and CFI measures the fit of a hypothesized model compared to a baseline model with the worst fit. Statistical analyses were performed using STATA 18 (StataCorp, College Station, TX, USA).

### 2.6. Statistical Analysis

Values are presented as means ± standard deviations (SD). Group comparisons were performed using the analysis of variance for continuous variables and the chi-square test for categorical variables. Due to the right-skewed distribution of MIS and AhRL, a log transformation was applied to these variables. The correlation coefficients between MIS and the parameters of metabolic or lung function were analyzed using the Pearson correlation test. Linear regression analyses evaluated the association of MIS and AhRL with body mass index (BMI) or lung function, with adjustments for age, sex, height, smoking, drinking, energy expenditure, and serum fasting glucose and triglyceride concentrations. We created four subgroups based on the levels of MIS_ATP_ and MIS_ROS_ and examined the differences in the correlations between lung function and BMI between these subgroups. *p* < 0.05 was considered statistically significant. Statistical analyses were performed using STATA 18 (StataCorp, College Station, TX, USA).

### 2.7. AI-Assisted Editing

We utilized ChatGPT-4 for English language editing to identify grammatical errors and enhance the clarity and readability of the manuscript. We did not use AI tools for data management or analysis; therefore, this process did not influence the data or alter the conclusions of our study.

## 3. Results

### 3.1. General Characteristics of Studied Population

The 1371 participants had a mean age of 60.9 ± 8.5 years, with 52.4% being women (Table 1). The mean BMI was 24.3 ± 3.2 kg/m^2^, with 41.3% of men and 58.7% of women classified as obese (BMI > 25 kg/m^2^, based on the Korean criterion for obesity). Among participants, 25.4% had diabetes, 15.9% had impaired glucose tolerance (IGT), and 58.7% had normal glucose tolerance (NGT). Additionally, 17.5% were current smokers and 44.2% were alcohol consumers. The unadjusted FVC and FEV_1_ were 3.41 ± 0.83 L and 2.62 ± 0.63 L, respectively. When participants were sub-grouped by BMI, the proportion of females increased, and all other clinical parameters, including AhRL, MIS, FVC, and FEV_1_, showed significant changes across BMI (*p* for trend < 0.01).

### 3.2. Pearson Correlation Analysis of AhRL and MIS with Clinical Parameters

MIS_ATP_ levels decreased with aging, while MIS_ROS_ levels increased (Table 2), both representing mitochondrial dysfunction. MIS_ATP_ is significantly correlated with MIS_ROS_ (r = −0.27, *p* < 0.001). In both sexes, MIS_ATP_ was correlated with obesity (waist circumference), insulin resistance (HOMA-IR, triglycerides), and diabetes (HbA1c, fasting blood glucose, insulin). Additionally, LDL-cholesterol and lung function (FVC, FEV_1_) were positively correlated with MIS_ATP_ in both sexes. In females, height, blood pressure (SBP, DBP), and obesity (weight, BMI) showed significant correlations with MIS_ATP_, whereas in males, they were not correlated with MIS_ATP_.

MIS_ROS_ exhibited significant positive correlations with obesity (waist circumference), insulin resistance (HOMA-IR), diabetes (HbA1c), fasting blood glucose, and insulin in both sexes. Unlike MIS_ATP_, weight, hip circumference, BMI, and triglycerides were positively correlated with MIS_ROS_ only in males, whereas SBP showed a significant correlation only in females. FVC showed a significant negative correlation with MIS_ROS_ only in males, while FEV_1_ did not show a significant correlation with MIS_ROS_ in both sexes. AhRL also showed significant negative correlation with FVC and FEV1, as well as metabolic parameters.

### 3.3. Sex-Dependent Associations of AhRL and MIS with BMI or Lung Function

Table 3 represents the multivariate linear regression analysis results for MIS and AhRL with BMI, or lung function, adjusted for age, height, smoking and drinking habits, and fasting blood glucose and triglyceride levels. MIS_ATP_ was significantly correlated with BMI only in females (β = −7.747, *p* < 0.001), while MIS_ATP_ was correlated with FVC only in males (β = 0.905, *p* = 0.009). In contrast, MIS_ROS_ showed a significant positive association with BMI (β = 4.365, *p* = 0.032) and a negative association with FVC in males (β = −0.819, *p* = 0.028), but not in females. This indicated that MIS affecting ATP production plays a significant role in female obesity, while MIS affecting ROS is more relevant to male obesity. Additionally, only in males, FVC-indicated lung capacity is significantly correlated with mitochondrial dysfunction, demonstrated by decreased MIS_ATP_ and increased MIS_ROS_. However, FEV_1_ did not show significant associations with either MIS_ATP_ or MIS_ROS_. AhRL showed significant association with BMI only in females. There were no significant associations between AhRL level and lung function parameters.

**Figure 2:**
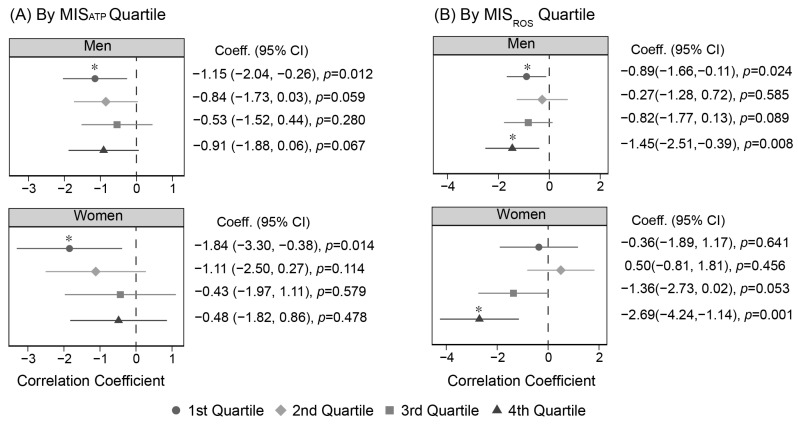
Correlation coefficient between FVC and BMI according to MIS-quartile subgroups. (**A**) Subgroups according to the quartile of MIS_ATP_; (**B**) Subgroups according to the quartile of MIS_ROS_. The points and bars represent the correlation coefficient and their respective 95% confidence intervals (CI) for FVC concerning the obesity parameter, as calculated through linear regression. * Indicates significant *p*-value. Each regression analysis was performed with adjustments for age, height, smoking, and drinking. BMI, body mass index; FVC, forced vital capacity.

To explore the correlation between FVC and BMI under the effects of MIS_ATP_ or MIS_ROS_, a correlation analysis was conducted in MIS-quartile subgroups. Figure 2A illustrates that in the 1st quartile of MIS_ATP_ (the subgroup with highest MIS), there was a significant negative association between BMI and FVC for both males and females. However, in the 2nd, 3rd, and 4th quartiles of MIS_ATP_, no significant association between FVC and BMI was observed in either gender. Figure 2B shows the association between FVC and BMI across MIS_ROS_ quartile subgroups. Similar to MIS_ATP_, the 4th quartile of MIS_ROS_ (the subgroup with highest MIS) also revealed a significant negative association between FVC and BMI in both male and female. In contrast, within the lowest MIS_ROS_ quartile (the 1st quartile), a significant association was found only in males, not in females. These results suggest that under the conditions of mitochondrial dysfunction, FVC-indicated lung capacity decreases as BMI (obesity) increases.

### 3.4. Structural Equation Modeling (SEM)

We developed the SEM model based on the assumption that mitochondrial function state is central to the association network. The latent variable ‘Mitochondrial Function’ was conceptualized as influenced by MIS levels. Given their statistical significance in association with MIS, BMI and FVC were selected as outcome variables for the SEM model. In our model, both MIS_ATP_ and MIS_ROS_ served as the measured variables representing the amount of these substances in the blood of participants. SEM analyses were conducted separately for each gender using the same model structure. The model showed satisfactory fit, with RMSEA values below 0.06 and CFI values exceeding 0.95, indicating reasonable goodness of fit.

**Figure 3:**
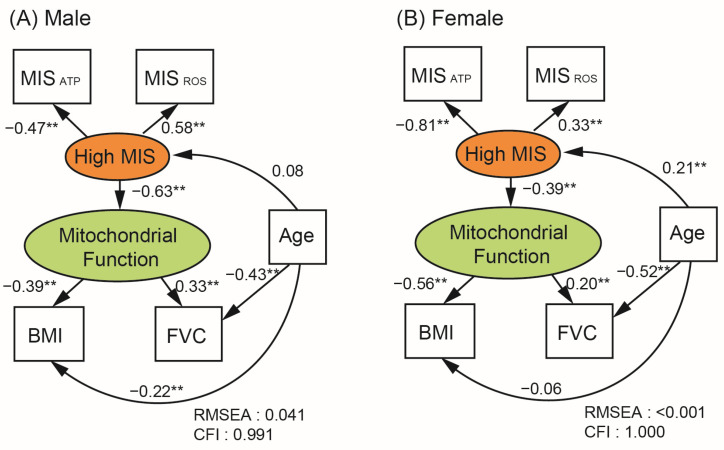
Structural Equation Model (SEM) of the framework of interaction among MIS, mitochondrial function, BMI, and Lung function. Variables in squares represent measured variables, while variables in ovals represent latent, unmeasured, and conceptual variables in our model. Numbers next to arrows indicate correlation coefficients between paired variables, with ** indicating statistical significance (*p* < 0.001). MIS_ATP_, mitochondrial inhibitor substance measured by intracellular ATP; MIS_ROS_, mitochondrial inhibitor substance measured by intracellular ROS; BMI, body mass index; FVC, forced vital capacity; RMSEA, the root mean square error of approximation; CFI, comparative fit index.

As shown in Figure 3, MIS levels were reflected by MIS_ATP_ (Coefficient = −0.47 for male, −0.781 for female, *p* < 0.001) and MIS_ROS_ (Coefficient = 0.58 for male and 0.33 for female, *p* < 0.001). Consequently, the latent variable ‘High MIS’ negatively influenced the ‘Mitochondrial Function’ (Coefficient = −0.63 for male, −0.39 for female, *p* < 0.001). In other words, MIS was negatively correlated with MIS_ATP_ and positively correlated with MIS_ROS_ in both sexes, and MIS was negatively associated with mitochondrial function. ‘Mitochondrial Function’, influenced by MIS, affected BMI (Coefficient = −0.39 for male, −0.56 for female, *p* < 0.001) and FVC (Coefficient = 0.33 for male, 0.20 for female, *p* < 0.001). Better mitochondrial function was associated with lower BMI and higher lung function. It confirms that, as mitochondrial function declined, BMI increased, and lung function decreased.

Since age could influence MIS and lung function, the age variable was incorporated in the model. Age showed a negative association with FVC in both sexes (Coefficient = −0.43 for male, −0.52 for female, *p* < 0.001). Age was positively associated with MIS in females, not in males. BMI decreased with age in males (Coefficient = −0.22 for males, *p* < 0.001), but no such association was observed in females. Therefore, the impact of age on BMI was considered an indirect effect mediated through MIS-mitochondrial function.

## 4. Discussion

This study presents several important findings. Serum MIS levels, as measured by MIS_ATP_ and MIS_ROS_, serve as indirect but quantitative indicators of mitochondrial function in subjects. Our results demonstrate that mitochondrial function, reflected by these serum biomarkers, is strongly associated with BMI, an obesity indicator, and more importantly, with FVC, an indicator of lung capacity. Notably, sex-dependent differences in the associations of BMI and FVC with mitochondrial dysfunction. These findings suggest that MIS-based serum biomarkers could be critical for assessing lung function and obesity in humans exposed to EPCs.

Mitochondria are essential for energy production and metabolism, and their dysfunction is linked to metabolic disorders such as obesity, diabetes, and insulin resistance [5,6,23]. However, measuring mitochondrial function in individuals, especially within large cohort studies, remains challenging. In 2003, Shulman’s group reported that mitochondrial oxidative and phosphorylation activity was reduced by approximately 40% in elderly individuals with insulin resistance as assessed by in vivo NMR spectroscopy [24]. However, in vivo NMR is impractical for determining the mitochondrial function of the participants in cohort studies or clinical settings. Simpler and more widely applicable mitochondrial function assays are needed to determine the relationship between mitochondrial function and metabolic disorder. Here, the present correlation analyses and SEM model demonstrated that MIS_ATP_ and MIS_ROS_ can serve as simpler, more widely applicable alternatives for assessing mitochondrial function.

We conceptualized MIS as encompassing all substances in the blood that influence mitochondrial function, including dioxins, dioxin-like chemicals, environmental pollutants, pharmaceuticals, and nutrients. The cell-based MIS_ATP_ and MIS_ROS_ assays offer a way to indirectly estimate the effects of these substances on mitochondrial function by measuring intracellular ATP and ROS levels in murine hepatoma cells treated with human serum. A wide variety of substances in blood can affect mitochondrial function in cultured cells, and effects can be inhibitory, stimulatory, or neutral. MIS therefore represents the biological sum of these interactions. In other words, MIS levels represent the overall “Mitochondrial Function” influenced by the presence of mitochondrial inhibitor substances, MIS. Our findings are consistent with previous research, which indicated that MIS_ATP_ is significantly associated with the risk of developing diabetes and to be a strong predictive factor of this disease [5]. We believe that these assays may replace in vivo NMR spectroscopy as a tool for assessing “Mitochondria Function” in individuals.

It is important to note that this study, being observational, shows only cross-sectional relationships between parameters rather than cause-and-effect relationships. Lung function is influenced by numerous environmental and individual factors. To assess the association of MIS and AhRL with lung function, we performed multivariate analyses, adjusting for potential confounders such as age, sex, height, smoking and drinking habits, fasting glucose, and triglycerides. Although the limitations of epidemiological studies do not allow us to fully account for unmeasured or uncollected factors in the cohort, the statistically significant effect of MIS but not AhRL on lung function suggests the association is reliable. The strong correlations observed between MIS, mitochondrial function, BMI, and lung function in our SEM analyses provide valuable insights into these complex interactions. When MIS and mitochondrial dysfunction were incorporated as latent variables, SEM analyses revealed statistical associations between MIS and mitochondrial function, as well as between mitochondrial function and BMI, or lung function. One of the strengths of this study is the use of serum samples from the well-established KoGES cohort with the consistent measurements of MIS and AhRL conducted at a single institution, ensuring highly reproducibility. A limitation, however, is that our assay systems have yet to be validated by other laboratories. Despite this, the predictive value of MIS for diabetes has been confirmed across diverse populations, including Swedish elderly individuals, Brazilians, and pregnant subjects in Korea [7,9,25]. Another limitation of our study is the absence of a healthy control group. Since the KoGES Ansung cohort represents the general population of rural farming communities, we could not determine a clear “normal” level of AhRL in pM TCDDeq. However, the AhRL in participants with normal glucose tolerance (NGT) was 1.72 ± 0.97 [5], which is slightly higher than the reported normal range (0.25~1 pM TEQ). Given that this cohort includes individuals with various health conditions and that AhRL reflects the total biological activity of AhR ligands, including dioxins and dioxin-like chemicals in serum, it is expected that the average TCDDeq value would be higher than the chemically determined “normal” level. In our multivariate correlation analysis, the lack of a significant association between AhRL and lung function suggests that only those dioxins and dioxin-like chemicals that specifically inhibit mitochondrial function are associated with lung function impairment.

There are several factors that can alter mitochondrial function. First, a high-fat diet increases free fatty acids, leading to an overload on the mitochondrial oxidative phosphorylation system. This overload results in ROS production and ultimately causes mitochondrial dysfunction. Second, chronic low-level inflammation, often associated with obesity and metabolic disorders, can trigger the production of inflammatory cytokines. These cytokines can disrupt the mitochondrial electron transport chain, increasing ROS and impairing mitochondrial function in muscle and liver tissues [23]. Third, mutations in mitochondrial DNA and aging are also implicated in mitochondrial dysfunction. Finally, exposure to multiple environmental pollutants, such as EDCs, heavy metals, and air pollutants, can induce mitochondrial dysfunction and elevate ROS production, which are closely associated with the development of diabetes and obesity [26,27,28].

Mitochondrial health may significantly influence lung function, reflecting whole-body energy metabolism. An age-progressive decline in lung function, typically marked by reductions in FEV1 and FVC, has been reported. At the cellular level, although knowledge is limited, altered mitochondrial homeostasis has been observed in the lungs of healthy aging individuals [11]. In aged lung cells, mitochondria become enlarged and fused, accompanied by increased mitochondrial ROS production and impaired respiration. Aging may lead to mitochondrial dysfunction in lung cells by reducing the expression of mitochondrial homeostasis regulators, potentially contributing to various lung diseases. Maladaptive responses to stress may exacerbate mitochondrial dysfunction, further affecting lung function [11]. Although clinical studies specifically examining lung function in the context of mitochondrial dysfunction are limited, associations between decreased lung function and obesity or diabetes have been reported [14,29,30]. Our study confirmed the correlation between lung function and obesity indices, particularly in the highest MIS subgroup (characterized by the lowest MIS_ATP_ and highest MIS_ROS_ levels). This finding suggests that MIS-mediated mitochondrial dysfunction may influence the relationship between lung function and obesity.

Pulmonary function declines with age, even in the absence of known lung diseases or risk factors such as smoking [31]. The interaction between MIS and lung function involves complex detrimental effects on mitochondrial health in both the lungs and the entire body. The CARDIA study highlights this interaction, showing that initially thin young adults experience increased lung function with rising BMI until age 38, after which higher obesity leads to significant declines in lung function [32]. These changes reflect adaptive responses to environmental stressors, a concept described by Sterling and Eyer as “allostasis” [33]. This adaptive process involves changes in hormone levels, immune functions, and neurotransmitter activity. However, dysregulated allostasis in response to a high “allostatic load” is associated with various health problems. When this model is expanded to include glucose imbalance or elevated glucose level, “mitochondrial allostatic load” more accurately describes the deleterious structural and functional changes in mitochondria that occur in response to stress-related pathophysiology [34]. We suggest that MIS-induced mitochondrial allosteric load connects mitochondrial dysfunction with obesity and the decline in lung function associated with aging.

Sex differences in the correlation of BMI with MIS_ATP_ or MIS_ROS_ levels are noteworthy. Obese men exhibit high MIS_ROS_ levels, while obese women show low MIS_ATP_ levels. These findings suggest that ROS plays a more significant role in inducing obesity in men, whereas mitochondrial oxidative phosphorylation has a greater impact on women. In contrast, both MIS_ATP_ and MIS_ROS_ were correlated with FVC, a measure of lung function, in men but not in women. Sex differences in lung function are well-established [10], arising from a combination of anatomical, physiological, and hormonal factors. The stronger effect of MIS on lung function in men may highlight the influence of sex hormones in regulating lung function. For instance, muscle-specific estrogen receptor α knockout mice exhibit obesity and skeletal muscle resistance and impaired mitochondrial respiratory function due to decreased mitochondrial DNA polymerase Polg1 [35]. Additionally, mitochondrial uncoupling protein UCP3 has been identified as an estrogen-repressed gene, suggesting that estrogen helps maintain efficient ATP production in mitochondria by suppressing energy dissipation. Furthermore, estrogen and androgen receptors are localized in mitochondria [36,37], playing key roles in regulating ROS or mitochondria biogenesis depending on the tissue types. In the presence of estrogen (in females), the MIS level may not be sufficient to disturb mitochondrial function in the lungs, which could explain the observed sex differences. This implies that endocrine disrupting chemicals (EDCs), particularly those that are anti-estrogenic or androgen-disrupting chemicals, may be involved in determining lung function [38].

## 5. Conclusions

In this observational study, MIS showed a positive association with BMI and a negative association with FVC, with significant BMI-FVC correlations observed in the highest MIS subgroup. Our SEM model, developed from our hypothetical framework, successfully demonstrated the relationships among MIS, mitochondrial function, BMI, and FVC.

## Figures and Tables

**Table 1:** Clinical and laboratory characteristics of this study participants.

Variables	Total	BMI < 25	25 ≤ BMI < 30	30 ≤ BMI	*p* for Trend
Number	1371	817	491	63	
Female (n/%)	718 (52.4%)	393 (48.1%)	276 (56.2%)	49 (77.8%)	<0.001
Age (years)	60.9 ± 8.5	61.4 ± 8.6	60.3 ± 8.3	60.5 ± 8.1	0.208
Height (cm)	159.1 ± 9.1	159.7 ± 8.9	158.7 ±9.4	154.9 ± 7.2	<0.001
Weight (kg)	61.7 ± 10.1	56.9 ± 7.9	67.8 ± 8.5	76.5 ± 8.3	<0.001
Waist circumference (cm)	88.5 ± 8.7	83.8 ± 6.6	94.5 ± 5.5	103.8 ±6.3	<0.001
Hip circumference(cm)	92.5 ± 5.4	89.6 ± 3.9	95.9 ±3.6	103.3 ± 4.5	<0.001
BMI (kg/m^2^)	24.3 ± 3.2	22.2 ± 1.9	26.8 ± 1.3	31.8 ± 1.5	<0.001
SBP (mmHg)	121.4 ± 16.0	119.8 ± 16.4	123.6 ± 15.1	125.2 ± 16.1	<0.001
DBP (mmHg)	75.9 ± 8.8	74.6 ± 8.8	77.7 ± 8.4	78.4 ± 8.3	<0.001
Smoking (n/%)					<0.001
Never	861 (62.9%)	486 (59.6%)	325 (66.2%)	50 (79.4%)	
Ex-	269 (19.6%)	156 (19.1%)	102 (20.8%)	11 (17.5%)	
Current	240 (17.5%)	174 (21.3%)	64 (13.0%)	2 (3.2%)	
Alcohol consumption (n/%)					<0.001
Never	677 (49.4%)	377 (46.2%)	259 (52.7%)	41 (65.1%)	
Ex-	87 (6.4%)	54 (6.6%)	27 (5.5%)	6 (9.5%)	
Current	606 (44.2%)	385 (47.2%)	205 (41.8%)	16 (25.4%)	
Diabetes mellitus (n/%)					<0.001
NGT	805 (58.7%)	521 (63.8%)	259 (52.7%)	25 (39.7%)	
IGT	218 (15.9%)	107 (13.1%)	98 (20.0%)	13 (20.6%)	
DM	348 (25.4%)	189 (23.1%)	134 (27.3%)	25 (39.7%)	
HbA1c (%)	5.87 ± 0.99	5.81 ± 1.05	5.92 ± 0.88	6.20 ± 1.02	<0.001
Fasting glucose (mg/dL)	103.3 ± 31.5	101.9 ± 34.1	104.6 ± 23.9	111.9 ± 44.2	0.004
Fasting insulin (μIU/mL)	9.72 ± 6.72	8.79 ± 5.71	10.73 ± 7.54	13.96 ± 9.07	<0.001
HOMA-IR	2.56 ± 2.39	2.29 ± 2.21	2.84 ± 2.50	3.94 ± 2.99	<0.001
Total cholesterol (mg/dL)	190.8 ± 33.6	188.2 ± 32.8	194.5 ± 34.3	194.6 ± 35.8	<0.001
LDL-cholesterol (mg/dL)	117.3 ± 30.7	115.8 ± 29.4	119.4 ± 32.5	120.1 ± 32.4	0.032
Triglyceride (mg/dL)	141.1 ± 85.3	130.4 ± 80.3	157.7 ± 92.4	149.2 ± 69.2	<0.001
AhRL (pM, TCDDeq)	2.73 ± 1.85	2.59 ± 1.76	2.89 ± 1.96	3.42 ± 1.79	<0.001
MIS_ATP_ (% Control)	89.16 ± 12.67	90.07 ± 12.35	88.19 ± 13.19	84.84 ±11.36	<0.001
MIS_ROS_ (% Control)	115.76 ± 15.47	115.11 ± 15.04	116.10 ± 15.92	121.64 ± 16.35	0.005
FVC (liters, unadjusted)	3.41 ± 0.83	3.50 ± 0.82	3.34 ± 0.83	2.90 ± 0.71	<0.001
FEV_1_ (liters, unadjusted)	2.62 ± 0.63	2.65 ± 0.62	2.61 ± 0.64	2.36 ± 0.58	0.005

Notes: Continuous variables are presented as mean ± standard deviation (SD), while categorical variables are expressed as numbers (percentage). *p* for trend was calculated by a linear-by-linear association test. BMI, Body Mass Index; SBP, systolic blood pressure; DBP, diastolic blood pressure; NGT, normal glucose tolerance; IGT, impaired glucose tolerance; DM, diabetes mellitus; HOMA-IR, Homeostatic Model Assessment for Insulin Resistance; AhRL, aryl hydrocarbon receptor ligand; MIS_ATP_, mitochondrial inhibitor substance measured by intracellular ATP; MIS_ROS_, mitochondrial inhibitor substance measured by intracellular ROS; FVC, forced vital capacity; FEV_1_, forced expiratory volume in 1 s.

**Table 2:** Correlation coefficients between MIS and metabolic or lung function parameters.

	MIS_ATP_				MIS_ROS_				AhRL			
	Men		Women		Men		Women		Men		Women	
	Coef.	*p*	Coef.	*p*	Coef.	*p*	Coef.	*p*	Coef.	*p*	Coef.	*p*
Age	−0.091	0.019 *	−0.163	<0.001 *	0.014	0.717	0.063	0.091	0.120	0.003 *	0.181	<0.001 *
SBP	−0.070	0.071	−0.178	<0.001 *	0.073	0.062	0.123	0.001 *	0.118	0.003 *	0.124	0.001 *
DBP	0.001	0.996	−0.083	0.026 *	−0.026	0.503	−0.001	0.963	0.012	0.769	0.064	0.088
Height	0.027	0.486	0.103	0.005 *	−0.045	0.242	−0.017	0.644	−0.038	0.328	−0.130	<0.001 *
Weight	−0.051	0.192	−0.099	0.008 *	0.107	0.006 *	0.050	0.183	0.071	0.072	0.040	0.294
Waist circumference	−0.125	0.001 *	−0.202	<0.001 *	0.186	<0.001 *	0.107	0.004 *	0.145	<0.001 *	0.164	<0.001 *
Hip circumference	−0.061	0.120	−0.068	0.067	0.094	0.017 *	0.023	0.538	0.080	0.046 *	0.012	0.749
BMI	−0.076	0.052	−0.165	<0.001 *	0.157	<0.001 *	0.065	0.083	0.108	0.006 *	0.122	0.001 *
HbA1c	−0.238	<0.001 *	−0.333	<0.001 *	0.208	<0.001 *	0.240	<0.001 *	0.425	<0.001 *	0.409	<0.001 *
FBC	−0.215	<0.001 *	−0.263	<0.001 *	0.294	<0.001 *	0.248	<0.001 *	0.343	<0.001 *	0.255	<0.001 *
Fasting insulin	−0.082	0.035 *	−0.141	<0.001 *	0.161	<0.001 *	0.143	<0.001 *	0.104	0.009 *	0.141	<0.001 *
HOMA-IR	−0.149	<0.001 *	−0.198	<0.001 *	0.235	<0.001 *	0.181	<0.001 *	0.194	<0.001 *	0.215	<0.001 *
Total cholesterol	−0.001	0.756	0.046	0.219	0.006	0.879	−0.069	0.062	−0.015	0.695	0.046	0.218
LDL cholesterol	0.084	0.032 *	0.074	0.046 *	−0.065	0.095	−0.069	0.064	−0.100	0.012 *	−0.019	0.627
Triglyceride	−0.196	<0.001 *	−0.140	<0.001 *	0.181	<0.001 *	0.064	0.087	0.191	<0.001 *	0.192	<0.001 *
FVC	0.148	<0.001 *	0.144	<0.001 *	−0.121	0.003 *	−0.077	0.058	−0.117	0.005 *	−0.129	0.001 *
FEV_1_	0.136	<0.001 *	0.154	<0.001 *	−0.079	0.054	−0.063	0.117	−0.126	0.002 *	−0.125	0.002 *

Notes: Correlation coefficients and *p*-values were calculated using Pearson’s correlation test. * Indicates a significant *p*-value. Values in gray indicate *p* > 0.05. MIS_ATP_, mitochondrial inhibitor substance measured by intracellular ATP; MIS_ROS_, mitochondrial inhibitor substance measured by intracellular ROS; AhRL, aryl hydrocarbon receptor ligand; Coef., correlation coefficient; SBP, systolic blood pressure; DBP, diastolic blood pressure; BMI, body mass index; FBC, Fasting blood glucose; HOMA-IR, Homeostatic Model Assessment for Insulin Resistance; FVC, forced vital capacity; FEV_1_, forced expiratory volume in 1 s.

**Table 3:** Multivariate analyses of the association of MIS with BMI or lung function.

	MIS_ATP_				MIS_ROS_				AhRL			
	Men		Women		Men		Women		Men		Women	
	Beta (SE)	*p*	Beta (SE)	*p*	Beta (SE)	*p*	Beta (SE)	*p*	Beta (SE)	*p*	Beta (SE)	*p*
BMI	−1.498 (1.848)	0.418	−7.747 (2.105)	<0.001 *	4.365 (2.029)	0.032 *	2.730 (2.314)	0.238	0.558 (0.392)	0.155	0.854 (0.378)	0.024 *
FVC	0.905 (0.344)	0.009 *	−0.062 (0.256)	0.807	−0.819 (0.371)	0.028 *	−0.318 (0.288)	0.270	−0.053 (0.072)	0.380	0.010 (0.044)	0.820
FEV_1_	0.582 (0.306)	0.058	0.035 (0.219)	0.872	−0.512 (0.331)	0.122	−0.148 (0.247)	0.546	−0.070 (0.054)	0.270	0.006 (0.038)	0.878

Notes: Beta-coefficients and *p*-values were calculated using multivariate linear regression for the correlation of MIS and AhRL with BMI or lung function, with adjustments for age, height, smoking, drinking, fasting glucose, and triglyceride levels. * Indicates significant *p*-value. Values in gray indicate *p* > 0.05. Beta, β-coefficient; SE, standard error; BMI, body mass index; MIS_ATP_, mitochondrial inhibitor substance measured by intracellular ATP; MIS_ROS_, mitochondrial inhibitor substance measured by intracellular ROS; AhRL, aryl hydrocarbon receptor ligand; FVC, forced vital capacity; FEV_1_, forced expiratory volume in 1 s.

## Data Availability

The datasets presented in this article are not readily available due to 3rd Party restrictions.
